# The Impact of Social Media on Adolescents’ Eating and Sleeping Habits: A Systematic Review and Meta-Analysis

**DOI:** 10.3390/healthcare13222962

**Published:** 2025-11-18

**Authors:** Alicia Cal-Herrera, Ariadna Corbella-González, Silvia Climent-Llinares, Olga I. Fernández-Rodríguez

**Affiliations:** 1 INPRO Research Group, European University Miguel de Cervantes, 47012 Valladolid, Spain; alcal@uemc.es (A.C.-H.); oifernandez@uemc.es (O.I.F.-R.); 2 Independent Researcher, 03001 Alicante, Spain

**Keywords:** adolescence, social media, eating disorders, sleeping difficulties

## Abstract

**Background/Objectives**: Social media has become a key activity in adolescents’ lives, with potential implications for their health and well-being. Because of this, the objective was to examine the influence of social media on the eating behavior and sleep quality of adolescents aged 13 to 18 years. **Methods:** The PubMed, Scopus, Proquest, Web of Science, and Cochrane Library databases were reviewed following the PRISMA protocol. The inclusion criteria for the studies were as follows: a sample of adolescents aged 13 to 18 years without a baseline clinical diagnosis and research objectives related to social media and its influence on eating behavior or sleep quality. A total of 24 articles were included at the end of the search. Due to heterogeneity in measurement formats, a single pooled analysis was not feasible. Instead, two partial random-effects meta-analyses of continuous outcomes were performed (sleep and eating behaviours). **Results:** Qualitative synthesis revealed consistent associations between problematic social media use, poor sleep quality, and disordered eating. The meta-analyses showed a small-to-moderate and statistically significant association on sleep quality (r = 0.36) while the pooled estimate for eating behaviours was imprecise and not significant (r = 0.35), reflecting the very limited number of eligible studies. **Conclusions:** Excessive social media use is associated with poorer sleep and eating outcomes among adolescents. These findings highlight the need for educational and preventive strategies promoting healthy digital habits and psychological well-being. This systematic review elucidates the implications of social media use for health promotion at this development stage.

## 1. The Importance of Social Media Use in Adolescent Health

Social media use significantly influences aspects of adolescents’ psychological and physical development, as well as the construction of their identity.

It is important to understand the relationship adolescents have with social media, as it can have both positive and negative effects on their mental and physical health. The positive effects are related to increased social connection, greater access to information, and the creation of spaces that offer the opportunity to share new experiences, while the negative effects are related to addiction, a loss of control, cyberbullying, sleep disorders, and social comparisons, which are especially linked to body dissatisfaction.

## 2. Introduction

In recent years, social media use has grown exponentially among adolescents, becoming one of the most common activities in their daily lives [1]. Platforms such as Instagram, TikTok, Snapchat, Facebook, and YouTube provide a space for socialization and identity building, where adolescents can both consume and create content [2].

Although the World Health Organization (WHO) defines adolescence as the period between 10 and 19 years of age [3], the age range from 13 to 18 years is often considered a critical stage for social and emotional development, during which adolescents become especially sensitive to social feedback, peer approval, identity formation and mental health problems [4].

Social media provides an environment where adolescents can explore and express their identity, interact with their peers, and participate in various social activities [5]. However, continuous interaction can also lead to overexposure and dependence on social validation, affecting young people’s self-esteem and emotional well-being [4].

It is well known that intensive use of social media by adolescents is associated with some negative consequences for health and development in adolescence. According to the WHO HBSC report, 11% of adolescents exhibit problematic social media use, characterized by symptoms such as addiction (difficulty controlling their use and abandoning other activities in their daily lives). The same report and various studies highlight other risk factors such as social comparison, cyberbullying, less sleep quality, increased anxiety, and depressive symptoms [2,6].

Given these dynamics, it is necessary to further investigate the risk factors associated with social media use. Constant exposure can affect self-esteem and body image perception, which can lead to eating disorders [7], as well as anxiety and stress, factors that negatively affect sleep quality [8].

Furthermore, considering that both eating and sleeping share similar behavioural and psychological responses to factors such as stress, major life changes or social difficulties, it is important to examine adolescents’ habits related to nutrition and sleep [9]. Disruptions in either domain are often interrelated, as emotional distress or stressful experiences can lead to irregular eating patterns or sleep disturbances [10].

Therefore, from a health promotion perspective, it is necessary to consider the impact of social media use on adolescents, as well as to understand its relationship with eating habits and sleep quality at this stage of life. Based on these premises, this study aims to conduct a systematic review of the current scientific literature with the objective of examining the influence of social media on the eating behavior and sleep quality of adolescents aged 13 to 18 years.

## 3. Methods

This research was based on a systematic review following the PRISMA protocol [11]. The search protocol was registered in PROSPERO on 18 July 2025 (registration number CRD420251107338).

### 3.1. Research Question and Study Review Process

The research question was formulated following the participant, intervention, and outcome (PIO) structure, as follows: “What is the influence of social media use on the eating behavior and sleep quality of adolescents aged 13 to 18?” The research process included three phases: the initial database search (1), document screening in Covidence (2), and analysis of studies that met the inclusion criteria (3).

### 3.2. Search Strategy

Searches were conducted in the following databases: PubMed, Scopus, Proquest, Web of Science, and Cochrane Library. The MeSH terms “adolescence,” “social media,” “eating behavior,” and “sleep quality” were used, along with the descriptors “eating” and “sleeping,” in combination with the Boolean operators ‘AND’ and “OR.” Time filters (2020–2025) or age filters (13 to 18 years) were used whenever this was an option within the search system. Specifically in PubMed, time filters were used to narrow down the results to the age range 13–18. Table 1 shows the search formulas plus the initial results.

### 3.3. Eligibility Criteria

Considering that social media platforms are highly dynamic and continuously evolving, all articles published between 1 January 2020, and 18 July 2025 were considered in order to capture the most recent and relevant evidence written in Spanish or English on the topic.

The eligible age range for inclusion was set between 13 and 18 years. However, studies including broader age ranges were also considered if they clearly reported separate findings for the adolescent subgroup or if their mean participant age fell within the 13–18 range, as these samples were deemed representative of the adolescent population.

Studies focusing on adolescents aged 13 to 18 years without a baseline clinical diagnosis (such as eating disorders or addiction to the Internet or social media) and that established research objectives related to social media and its influence on eating behavior or sleep quality were taken into account. While studies that included children or adults without separating the sample by age, that weren’t focused on the influence of social media use on eating behavior or sleep quality, or that had a sample of adolescents who were athletes or had psychiatric disorders or chronic diseases were excluded.

Regarding the type of study, qualitative studies and other systematic reviews or meta-analyses were excluded from this review. Qualitative articles were not considered relevant to the specific research question, as they did not provide quantitative data applicable to the outcomes of interest. In the case of systematic reviews or meta-analyses, their reference lists were examined to identify original studies meeting our inclusion criteria, and only those primary articles were incorporated when appropriate.

### 3.4. Methodological Quality Assessment

The methodological quality of the included studies was independently assessed by two reviewers using the Joanna Briggs Institute checklists (JBI), with disagreements being resolved by a third reviewer [12]. The Risk of Bias in Non-randomized Studies Version 2 (ROBINS-I V2) tool was employed in order to evaluate the risk of bias at review level. This assessment allows for a consistent evaluation across different domains: study eligibility, identification and selection, data extraction and quality appraisal, as well as the synthesis and reporting of results [13]. The tool was applied independently and using a double-blind procedure.

### 3.5. Quantitative Synthesis (Meta-Analysis)

A single pooled meta-analysis was not feasible due to variability in the measurement instruments and outcomes across studies. Therefore, in addition to the qualitative synthesis, two partial random-effects meta-analyses were conducted to quantify the association between social media use and adolescent health outcomes.

Analyses were conducted by grouping studies into two domains (sleep and eating), only for studies that reported continuous outcomes allowing the computation of correlation coefficients. All analyses were performed using JASP (version 0.19, University of Amsterdam) [14], under the Classical Meta-Analysis module, applying a Der Simonian–Laird random-effects model.

Effect sizes were expressed as Pearson’s r, and correlations for analysis were Fisher z-transformed and back-transformed for interpretation. Effect sizes were extracted directly from the original studies or derived from verifiable numerical results following standard meta-analytic procedures.

Heterogeneity was assessed using Cochran’s Q, τ^2^, and I^2^ statistics, and publication bias was explored through residual funnel plots and leave-one-out diagnostics.

## 4. Results

**Figure 1:**
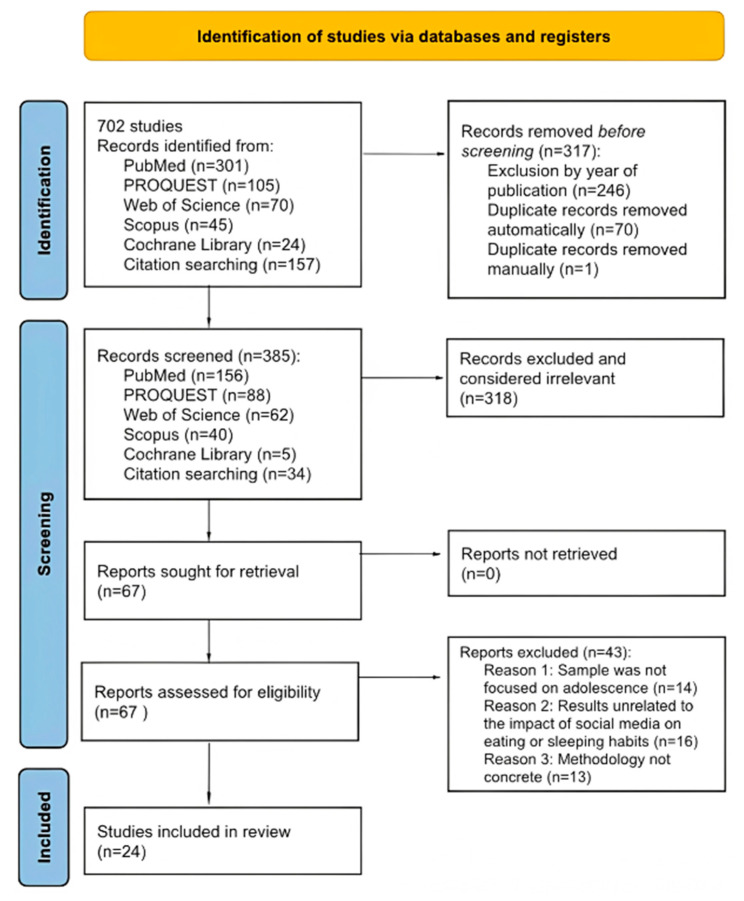
Flow diagram for systematic reviews. Note: Resource extracted from Page MJ et al. [15]. BMJ 2021;372:n71. doi: 10.1136/bmj.n71. This work is licensed under CC BY 4.0. To view a copy of this license, visit https://creativecommons.org/licenses/by/4.0/ URL (accessed on 22 July 2025).

Throughout the process, decisions regarding the inclusion or exclusion of studies were verified by two members of the research team. A total of 702 records were identified through database searches. Out of this initial number, 246 studies were excluded for having been published more than five years ago and 71 duplicates were identified. This resulted in 385 articles being examined by title and abstract, of which 318 studies were excluded. Finally, 67 full-text articles were analysed, of which 43 were excluded for the following reasons: having a child or adult sample (*n* = 14), not focusing on the impact of social media on eating habits or sleep quality (*n* = 16), or not having a methodology design appropriate for the research objective (*n* = 13). More detailed information on this process is presented in the PRISMA flow diagram (Figure 1).

### 4.1. Data Extraction and Quality Assessment

Table 2 and Table 3 show the characteristics of the 24 studies included in this study. Of the 24 studies, 9 investigated the impact of social media on eating habits [16,17,18,19,20,21,22,23,24], while 14 focused on sleep quality [25,26,27,28,29,30,31,32,33,34,35,36,37,38] and only 1 study included both health factors [39]. The tables represent these findings in a separate manner, classified by studies that focused on eating behaviours (Table 2) and studies that focused on sleep quality (Table 3). The study that included both health factors appears on both tables with separate findings.

**Figure 2:**
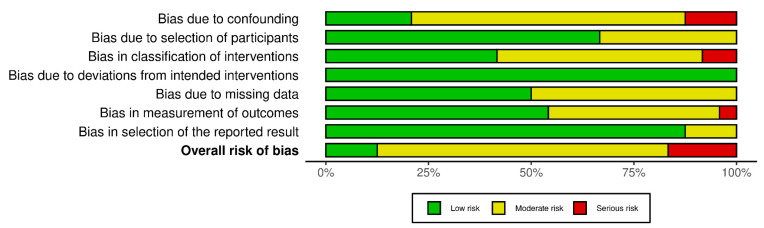
Risk of bias assessment. Note: This figure was produced employing the ROBINS-I V2 assessment tool [13].

Using the ROBINS-I V2 tool, an overall consensus of 85.11% was reached among the three reviewers, with most studies being rated as having a moderate overall risk of bias [13] (Figure 2).

### 4.2. Qualitative Synthesis: Social Media Use and Platforms Effect on Health

During adolescence, social media use is higher than among the adult population [16]. In some cases, it accounts for more than half of the day [17] making it very difficult to limit time spent on these platforms [18].

Khan et al. conducted a study involving 40 countries and found that all participants used social media, with women using it more frequently [20]. Meanwhile, Maksniemi et al. found that the younger the adolescents, the greater the difficulties in managing time online, suggesting that this population is particularly vulnerable in their social media use [38].

Several studies found specific relationships that affect health associated with the use of particular social platforms: TikTok use is associated with lower well-being, reduced academic participation, and procrastination in going to sleep [21], night time use of YouTube affects sleep quality [22], intensive use of Instagram causes eating problems [23], and use of Facebook or Snapchat causes sleep disturbances [18,24]. However, the main factor determining the impact of social media use on health is not related to the platform, but the content to which the adolescent is exposed [24,39]. Therefore, it is essential to analyze the content that is viewed within each social platform.

#### 4.2.1. Impact of Social Media on Eating Habits

The studies included emphasize that social media influences eating habits through multiple factors. Among the most common associations, it has been observed that social media use encourages more restrictive eating behaviours such as avoiding new foods, reducing or eliminating fruit and vegetable consumption, avoiding foods due to fear of expiration dates, and skipping meals [25,26]. Likewise, social media use has been linked to increased consumption of less healthy foods, preferences for fast food, and high consumption of sugary drinks, soft drinks, or caffeine [26,39].

Multiple studies have found an association between social media use and a higher likelihood of suffering from eating disorders [16,23,31,32,33,34], being more relevant in secondary school students than in adult populations [16]. No differences have been found with regard to gender, as both are affected similarly [31,34], although some studies have found a higher probability in women [22], and others in men [16].

#### 4.2.2. Impact of Social Media on Sleep Quality

Problematic and continuous use of social media has been associated with poorer sleep quality [18,20,21,22,24,35,36,37,38]. In addition, these platforms were associated with shorter sleep duration, nightmares, insomnia, difficulty falling asleep, and going to bed later [17,27,36]. Adolescents’ daily functioning was also affected, manifesting as daytime sleepiness, fatigue, emotional exhaustion, behavioural and emotional problems, conflicts with peers, and attention difficulties [19,38].

#### 4.2.3. Risk and Protective Factors for Social Media Use in Adolescence

In addition to analysing the prevalence and types of problems associated with social media that relate to eating and sleeping problems in adolescents, the included studies highlight that the origin of risk and protective factors is multifactorial, encompassing personal, social, and emotional aspects.

#### 4.2.4. Risk Factors for Social Media Use

Firstly, heavy use of social media was described as the most worrying risk factor in adolescents [18,20,21,23,26,31]. Although some studies conclude that time spent online is not associated with problems that interfere with adolescents’ daily lives [20,37], frequent use of these platforms is considered the beginning of problematic behavior, so the amount of time spent using them should be considered a warning sign [26,28,36].

Secondly, the social environment is considered another important factor [22,38,40]. Poor relationships with family/friends that encourage social comparisons increase the likelihood of health problems related to social media use [23,29]. Similarly, low self-esteem is associated with both sleep and eating problems [28,29,32].

In eating disorders, these risks include: obsessive thoughts about physical appearance [29,34], the need to edit photos before posting them [16], restrictive diets inspired by online content, overexposure to unhealthy eating information [33,39], and social interaction dependence through social media platforms [33].

With regard to sleep, the associated risk factors are: not turning off mobile devices before bed [38], high use of social media during day and night [17,24], and poor academic performance, which could be considered both a cause and a consequence of problematic social media use [21,30].

#### 4.2.5. Protective Factors for Social Media Use

To prevent harmful use of social media from affecting eating and sleeping habits, it is essential to promote a series of protective factors mentioned in the studies:

Firstly, it’s recommended to use social media platforms and the Internet for less than 5 h a day [29,36], avoiding night time use and establishing parental restrictions [37]. Secondly, training in emotional management is recommended, as continued use of social media can cause symptoms of anxiety and stress and the need to be constantly connected, so it is essential to learn to control these feelings to facilitate participation in other activities [31]. Thirdly, acquiring adequate digital skills will promote the ability to self-regulate the content consumed on social media, as well as self-reflection and solid self-esteem [29,32], which can reduce the presence of eating and sleeping problems by acquiring the ability to separate the digital world from the personal world [19,31]. Finally, promoting a quality social environment in which adolescents feel supported and can participate in various activities will reduce harmful use of social media [33,38].

### 4.3. Quantitative Synthesis: Meta-Analysis Results

A single pooled meta-analysis was not feasible due to substantial variability in measurement instruments and outcome formats across studies. Therefore, two partial random-effects meta-analyses were conducted to quantify the association between social media use and adolescent health outcomes in the domains of sleep and eating behaviours, using only studies that reported continuous outcomes from which correlation coefficients could be derived.

- **Eating (continuous outcomes):** Jaruga-Sękowska, 2025 [16]; Erdoğdu Yıldırım, 2025 [21].
- **Sleep (continuous outcomes):** Azhari et al. (2022) [34]; Miedzobrodzka et al. (2024) [33]; Bergfeld & Van den Bulck (2021) [35]; Hà et al. (2023) [32]; Makhfudli et al. (2020) [29].

**Figure 3:**
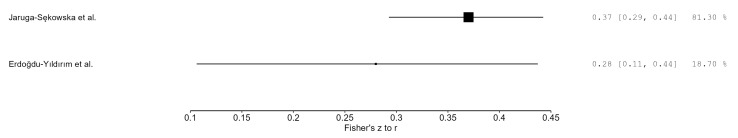
Forest plot of correlations between social media use and disordered eating indicators (random-effects model) [16,21]. Note: Each square represents an individual study, and the diamond indicates the pooled effect size (r), with 95% confidence intervals.

In the eating behavior domain, the random-effects model showed a pooled correlation of r = 0.35, with a wide confidence interval (95% CI: –0.13 to 0.70, *p* = 0.067), indicating no statistically significant association between social media use and eating-related outcomes. Between-study heterogeneity was not detectable (Q(1) = 0.97, *p* = 0.326; I^2^ = 0%), although the precision of these estimates is limited due to the very small number of included studies (Figure 3). 

**Figure 4:**
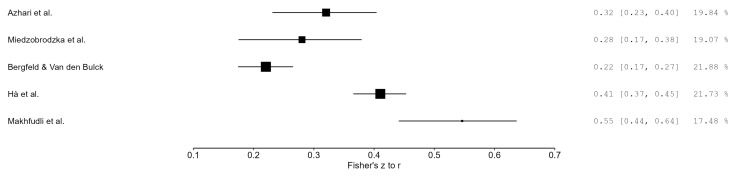
Forest plot of correlations between social media use and sleep quality (random-effects model) [29,32,33,34,35]. Note: Each square represents an individual study, and the diamond indicates the pooled effect size (r), with 95% confidence intervals.

Regarding sleep quality, higher social media use was moderately associated with poorer sleep quality across the five studies reporting continuous sleep indicators. The meta-analysis yielded a pooled effect of r = 0.36, 95% CI: 0.19 to 0.51, *p* = 0.005, with high heterogeneity (I^2^ = 92.07%) (Figure 4).

**Figure A1:**
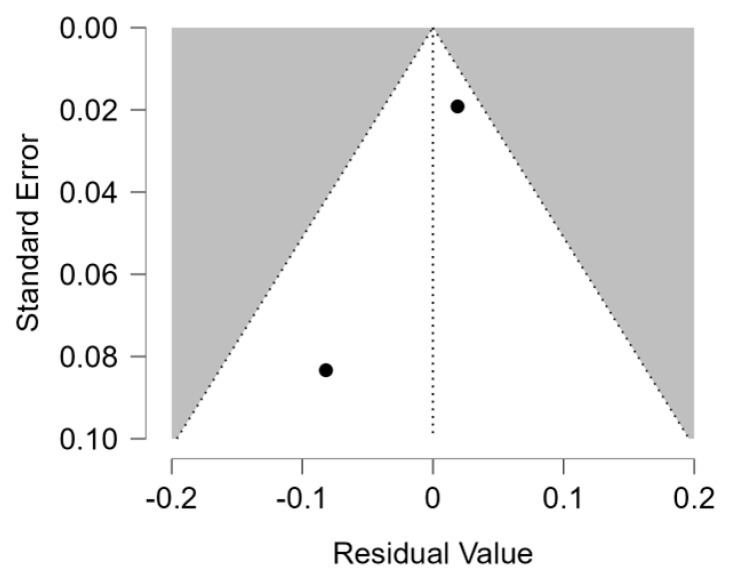
Residual funnel plot for meta-analysis of correlations between social media use and disordered eating indicators. Note. Each point represents an individual study; no marked asymmetry was observed.

**Figure A2:**
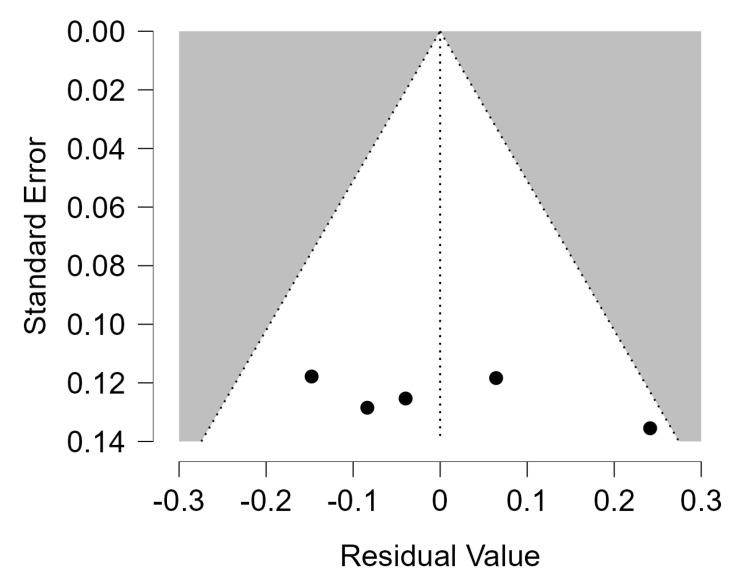
Residual funnel plot for the meta-analysis of correlations between social media use and sleep quality. Note. Each point represents a study; the vertical line corresponds to the pooled effect size, and the dashed lines indicate pseudo 95% confidence limits.

These partial meta-analyses provide quantitative support for the direction and magnitude of the associations identified in the forementioned qualitative synthesis. Residual funnel plots for each partial meta-analysis are provided in the Figure A1 and Figure A2.

Detailed results, including prediction intervals, τ^2^, and I^2^ values, can also be found in the Table A1 and Table A2.

## 5. Discussion

Adolescence is a crucial stage in development, characterized by a multitude of physical, psychological, and social changes that can have lasting repercussions on health and well-being. When problems arise during this period and are not addressed appropriately, the transition to adulthood can be compromised. In this context, it is essential to analyse the factors that influence adolescent health, among which the use of social media has become increasingly relevant [41,42].

The present review aimed to answer the following question: “What is the influence of social media use on the eating behavior and sleep quality of adolescents aged 13 to 18 years?”. The results indicate that social media use represents a challenge in intervention with major implications for the health and well-being of adolescents. The quantitative synthesis included only analyses based on continuous correlations due to heterogeneity in how outcomes were reported across studies. The meta-analysis of sleep quality revealed a small-to-moderate and statistically significant association, suggesting that more frequent or intensive social media use is reliably linked to poorer sleep quality across diverse adolescent samples. This result aligns with theoretical expectations related to sleep displacement, emotional arousal, and nighttime screen exposure, and indicates that sleep may be one of the most consistently affected health domains.

In contrast, the evidence regarding eating-related outcomes was much more limited. Although the pooled effect size was numerically similar, the confidence interval was wide and included zero, and only two studies met criteria for inclusion. Consequently, this association cannot be interpreted as reliable or robust. Instead, these results highlight a promising but currently inconclusive pattern that requires further research with standardized measures and larger samples.

Together, these findings suggest that while sleep disruptions appear to be a more firmly established correlate of adolescent social media use, the relationship with eating behaviours remains preliminary. Future studies should aim to harmonize measurement approaches and report sufficient statistical details to enable more comprehensive meta-analytic evaluations.

On the other hand, on the qualitative synthesis, evidence from large-scale population-based studies corroborates the patterns observed in smaller-scale investigations. For instance, both pan-European and Japanese studies involving tens of thousands of participants report associations consistent with those found in smaller, school-based or regional samples, suggesting that the observed relationships are not merely artifacts of limited sample sizes or local contexts [26,36].

Moreover, potential cultural differences should be acknowledged. Across diverse countries, girls generally report slightly higher social media use than boys, along with somewhat greater sleep-related difficulties, characteristics that may reflect broader sociocultural norms and gendered expectations surrounding technology use and well-being and that may have become more prevalent since the collective increase in mental health problems in adolescents of these last few years [43].

Nevertheless, it is important to consider that the observed associations between social media use, sleep quality, and eating behaviours may be partially influenced by additional variables that were not consistently controlled across studies. Several studies did not adjust for relevant confounders such as anxiety, depressive symptoms, BMI, socioeconomic status, parental supervision, or nighttime screen exposure, all of which are known to affect sleep and eating patterns in adolescents. In addition, the included studies were conducted in diverse cultural contexts across Asia, Europe, the Middle East, and South America, where cultural norms regarding body image, family routines, and technology use differ. Therefore, contextual and psychosocial factors may modulate the strength of the association. However, despite variability in confounding control and cultural context, all studies consistently showed the same direction of association.

Although the use of social media can have a positive impact on adolescence by facilitating social contact, promoting independence in seeking information, and developing creativity [44], the main finding of this systematic review was that social media affects eating habits and sleep quality due to factors such as time spent using it, platform content, and social influence.

### 5.1. Eating Habits

In relation to food, other studies have confirmed that multiple factors, such as food price, taste, product availability, social context, peer group relationships and influence, and exposure to online content, condition the acquisition of eating habits [45]. Intensive and continuous use of social media has been associated with low self-esteem, increased social comparison, and pressure to acquire an idealized body image, sometimes through the use of filters or digital retouching [11]. These dynamics favour the emergence of risky eating behaviours, which could lead to the development of an eating disorder, as well as the development of unhealthy eating habits such as skipping breakfast, consuming sugary drinks and fast food, and reducing vegetables and fruit intake [21,22,23,24,25,26,27].

Taking into account the impact social media has on eating habits, some recommendations in order to promote adolescents’ health could be as follows:

- Maintain regular meal times and avoid eating in front of screens.
- Involve adolescents in food preparation to strengthen their connection to real food.
- Promote a balanced diet rich in fruits, vegetables and proteins, avoiding excessive consumption of ultra-processed foods or caffeinated drinks.
- Recognition of the emotional impact of digital content (social comparisons, likes, beauty filters, etc.).

### 5.2. Sleep Quality

Sleep has been extensively studied, concluding that adequate rest is beneficial for strengthening cognitive skills and emotional well-being in adolescence [46]. However, it has been shown that sleep quality is poorer when social media is used at night, as well as when it is used under pressure to stay constantly connected [17,24]. This situation affects academic performance, causes emotional exhaustion and daytime sleepiness, thus increasing the risk of mental health problems [47].

Considering the impact social media has on sleep quality, some recommendations in order to promote adolescents’ rest are:

- Set a “disconnection hour” at least 30–60 min before bed without screens.
- Establish a consistent sleep routine leaving the phone outside the bedroom or in airplane mode overnight.
- Replace night scrolling with soothing activities (reading, listening to soft music, breathing exercises).
- Identify whether they wake up due to notifications or feel compelled to check social media.

### 5.3. Recommendations for Health in Adolescence

The implications of these findings are wide-ranging. On the one hand, they confirm that continued use of social media can be a significant risk factor for adolescents’ overall health, hindering participation in other activities and the formation of social bonds. Studies agree that adolescents’ vulnerability increases due to high exposure to content and social exposure online [48,49]. 

From a clinical perspective, these results suggest a series of protective factors and recommendations for health promotion, including age restrictions for access to platforms, parental controls on mobile devices, digital education for adolescents, and the importance of community spaces that encourage activities to strengthen personal and interpersonal relationships outside online platforms.

In the educational and healthcare fields, professionals should pay attention to how adolescents use social media to obtain early indicators of possible eating and sleeping disorders.

Some actionable steps that could be taken in order to address this social problem from a preventive approach could be:

1. Regulation and control access to social media: implementing measures that encourage responsible and conscious use of social media.
2. Promotion of healthy eating and sleeping habits: educating adolescents about self-care and helping them understand the importance of nutrition and rest.
3. Digital education and emotional literacy: covering aspects such as developing critical evaluation skills of online information or online safety limits.
4. Encouraging healthy community and social spaces: coordinating efforts to create healthy environments that reinforce a sense of belonging, cooperation and well-being.
5. The role of professional support: mental health and primary care professionals, along with families, play a key role in the early detection of problematic social media use. Some warning signs to watch for include sudden weight changes or excessive concern with appearance for eating habits and frequent complaints of tiredness or poor academic performance for sleep quality.

For this reason, it is recommended that prevention programs be implemented in schools, colleges, universities, health centers, community spaces, leisure and sports clubs, etc., including activities related to digital literacy, emotional self-regulation, and strengthening self-esteem.

With regard to public policy, the results obtained point to the importance of including awareness campaigns on the risks of excessive use of social media and the impact of different types of online content on adolescents.

Finally, as possible lines of future research, the need to conduct more in-depth longitudinal studies to explore differences between platforms and content over time has been identified, as well as preventive interventions aimed at strengthening digital health in adolescence.

## 6. Limitations

Most of the included studies have a cross-sectional design (*n* = 20), which may influence our understanding of the impact of social media use on eating habits and sleep quality in adolescents over time. The inclusion of additional databases, the review of grey literature, or the expansion of the search to encompass studies published in a broader range of languages could further improve the scope and comprehensiveness of this systematic review.

Although two partial meta-analyses were performed, it was not possible to include all studies in a single quantitative synthesis due to the diversity of measures and outcomes. Moreover, the number of studies included in each partial meta-analysis was small (2–4 studies), which limits the precision of the pooled estimates and the assessment of publication bias. Only studies reporting continuous outcomes that allowed the computation of correlation coefficients were included quantitatively. Several potentially relevant studies could not be incorporated into the meta-analyses due to heterogeneity in outcome formats or lack of extractable numerical data, which may reduce the comparability of findings.

Moreover, high heterogeneity was observed in the sleep meta-analysis, indicating that results may vary meaningfully depending on the operationalization of social media use and sleep constructs. The very small number of included studies, especially in the eating domain, also limits the assessment of publication bias: with only two studies, funnel plots and influence diagnostics cannot be reliably interpreted, and even in the sleep domain, high heterogeneity complicates their evaluation.

While self-reported measures were used across studies, many relied on validated standardized instruments (e.g., EAT-26, SCOFF, PSQI), which reduces measurement bias. However, control of confounding variables was inconsistent. Some studies adjusted for relevant covariates (e.g., age, sex, BMI, socioeconomic status, mental health), whereas others performed only bivariate analyses without multivariable adjustment. As shown in Supplementary Table S1, important confounders such as parental supervision, nighttime screen use, and psychological distress were frequently not considered, which may lead to an over- or underestimation of the true associations.

Furthermore, studies were conducted across heterogeneous cultural contexts (Asia, Europe, Middle East, North and South America). Cultural norms regarding social media use, body image, family routines, and sleep hygiene differ substantially, which may moderate the strength and direction of the associations and limit cross-context generalizability.

## 7. Conclusions

Problematic social media use during adolescence poses a significant challenge to mental health and well-being at this stage of development. The studies reviewed provide a comprehensive overview of the risk factors, impact, and consequences of using these platforms during adolescence.

While further research into this phenomenon is essential in order to design more effective intervention strategies tailored to the specific characteristics of different groups of adolescents, the relationship between social media and eating disorders or sleep quality should be considered a priority area for research and preventive interventions. Addressing these issues could help mitigate the long-term negative effects on young people’s health. The results from the meta-analyses specially show the need for more research focused on how social media affects eating behaviours. 

## Figures and Tables

**Table 1:** Search formulas.

Database	Search Strategy	Items Found
PubMed	(social media [Title/Abstract]) AND (eating behaviour [Title/Abstract]) OR (sleep quality [Title/Abstract])	301
Cochrane Library	“adolescence” in Title Abstract Keyword AND “social media” in Title Abstract Keyword AND “eating behaviour” in Title Abstract Keyword OR “sleep quality” in Title Abstract Keyword	24
Scopus	“adolescence” (Article title, Abstract, Keywords) AND “social media” (Article title, Abstract, Keywords) AND “eating behaviour” (Article title, Abstract, Keywords) OR “sleep quality” (Article title, Abstract, Keywords)	45
PROQUEST	abstract (adolescence) AND abstract (“social media”) AND abstract (eating) OR abstract (sleep)	105
Web of Science	“social media” AND “adolescence” AND “eating” OR “sleep” (Abstract)	70
Total		545

Note: Duplicate studies were subsequently identified automatically using the Covidence platform.

**Table 2:** Main characteristics of the selected studies that focused on eating behaviours. (n = 10).

First Author and Reference	Year	Country	Study Design	Goal	Population	Tools	Results
(Jaruga-Sękowska et al., 2025) [16]	2025	Poland	Cross-sectional	Examine the prevalence of eating disorder risk, eating behaviours, and self-esteem among people aged 16 to 25, as well as compare by age group and analyze the influence of social media on eating habits.	275 participants, including 113 high school students Age range 16–25 years, grouping high school students with a mean age of 16.7 (SD = 1.11) years, 63 females (55.7%) and 50 males (44.3%)	Sociodemographic and social media usage questionnaire, using the Eating Attitudes Test (EAT-26), Rosenberg Self-Esteem Scale (SES), and My Eating Habits (MEH) assessment tools.	High school students spent more time on social media High school students were at greater risk of developing an eating disorder (56.6%) than the group of university students or workers
(Erdodu Yildirim et al., 2025) [21]	2025	Turkey	Cross-sectional	Analyze the factors that predict the risk of developing an eating disorder in adolescence based on gender.	183 adolescents Average age of 15.65 (SD: 0.89) years, age range from 14 to 18 60.1% female and 39.9% male	Social Media Attitude Scale (SMAS), Eating Disorder Examination Scale (EDES), Eating Attitudes Test (EAT)	The use of social media anticipates problems in eating behavior because it causes greater stress and anxiety in adolescents Men were at greater risk of developing pathological eating behaviours due to social media
(Joo et al., 2024) [20]	2024	Korea	Cross-sectional	Examine the relationship between viewing culinary content on social media and the consumption of fast food, soft drinks, and caffeine among adolescents.	50,451 students Grades 7–9: 53.8% Grades 10–12: 46.2% 51% male and 49% female	Questions about viewing culinary content and consuming fast food, soft drinks, and caffeine	Viewing culinary content was associated with the consumption of fast food (male: OR:1.37; female: OR:1.46), soft drinks (male: OR:1.42; female: OR:1.51), and caffeinated beverages (male: OR:1.30; female: OR:1.24) among Korean adolescents. Viewing culinary programs more than once a week is associated with lower consumption of healthy foods.
(Livet et al., 2024) [22]	2024	Canada	Longitudinal	Examine vulnerability factors over a 5-year period in relation to screen use frequency and the severity of ED symptoms in Canadian adolescents.	3.801 adolescents Start: average age of 12.80 (SD = 0.40) years, ranging from 13 to 17 years	Three questions about screen use in terms of frequency, Three items of Developmental and Well-being Assessment ED section	Over the years, the risk of eating disorders increased among adolescents. Girls were more likely to have eating disorder symptoms. Adolescents who had greater exposure to social media had more eating problems and lower self-esteem.
(López-Gil et al., 2024) [39]	2024	Spain	Cross-sectional	Examine whether social media use and addiction are associated with eating disorders in Spanish adolescents	653 adolescents Average age of 14.0 years, ranging from 12 to 17 years old 44.0% boys and 56% girls	Addictive social media behaviours (SNAddS-6S), eating disorders (SCOFF), and questions about sleep duration	High social media use was associated with a greater likelihood of disordered eating behavior (odds ratio [OR] = 1.88, 95% confidence intervals [CI] 1.17–3.02), especially on Instagram.
(Zimmer-Gembeck et al., 2023) [19]	2023	Australia	Longitudinal	Identify risk factors measured in early adolescence (early risk factors, ages 10 to 13) for concern about online appearance measured five years later (ages 15 to 18).	261 Australian adolescents Aged 10 to 13 years at T1, 48% male; mean age 12.00 SD = 0.89 years	18 items from the Social Media Appearance Preoccupation Scale (SMAPS), Attitudes and Behavior Scale (ABS), three items from the Teasing Perception Scale, eight items from the Perceived Sociocultural Pressure Scale (PSPS), and objective BMI data.	Teenagers who feel judged by others for their appearance are more likely to use social media for aesthetic and obsessive purposes related to appearance and social comparisons.
(Jeong & Shin, 2022) [17]	2022	Republic of Korea	Cross-sectional	Analyze the relationship between adolescents’ use of social media and their eating behavior and satisfaction.	622 adolescents Average age of 16.1 (SD = 1.5), range from 10 to 19 years old 52.1% males and 47.9% females	KREI database “Consumer behavior survey for foods” in Korea. Questions on frequency of network use and satisfaction with eating habits and food consumption behavior.	Teenagers who used social media preferred to eat fast food and avoided fruits and vegetables, but this led to lower satisfaction with their diet.
(Ryu et al., 2022) [18]	2022	Republic of Korea	Cross-sectional	Examine associations between duration and type of smartphone content use with dietary risk factors in adolescents	54,601 adolescents (26,928 boys and 27,673 girls) The average age was 15.1 years (range: 12–18 years)	Self-report on duration and type of social media content. Dietary factors assessed: Skipping breakfast ≥5 times/week. Consumption of fruits, vegetables, instant noodles, fast food, snacks, and sugary drinks.	The use of smartphones for messaging/email, social media/forums, and gaming was associated with: - Lower consumption of fruits and vegetables. - Higher consumption of sugary drinks. - Greater likelihood of skipping breakfast. - Higher consumption of instant noodles.
(Frieiro et al., 2022) [23]	2022	Spain	Cross-sectional	Assessing the relationship between socializing through social media and the risk of developing eating disorders in Spanish high school students	721 secondary school students Average age of 13.89 (SD = 1.37) years with an age range of 12 to 18 years. 49.1% female	ESOC-39, Eating Attitudes Test-26 (EAT-26)	The use of social media is associated with wanting to go on diets. Frequent socializing through social media carries a higher risk of developing symptoms of eating disorders.
(Lonergan et al., 2020) [24]	2020	Australia	Cross-sectional	Examine whether behaviours related to photo-based social networks are associated with a higher likelihood of meeting criteria for eating disorders, and whether gender moderates this relationship.	4209 adolescents Boys (47%): 12–18 years old (M = 15.03, SD = 1.53) Girls (53%): 11–19 years old (M = 14.92, SD = 1.53)	EDE-Q, NEQ, DSM-5, K10, PedsQL SF15 criteria	All social media behaviours were associated with a higher likelihood of meeting criteria for Eds (χ^2^ [42] = 1128.93, *p* < 0.001). Fixation on aspects of other people’s photos was the most consistent predictor.

Note: BMI: Body Mass Index, ED: Eating Disorder.

**Table 3:** Main characteristics of the selected studies that focused on sleep quality (n = 15).

First Author and Reference	Year	Country	Study Design	Goal	Population	Tools	Results
(López-Gil et al., 2024) [39]	2024	Spain	Cross-sectional	Examine whether social media use and addiction are associated with eating habits in Spanish adolescents and how they relate to sleep	653 adolescents Average age of 14.0 years, ranging from 12 to 17 years old 44.0% boys and 56% girls	Addictive social media behaviours (SNAddS-6S), eating disorders (SCOFF), and questions about sleep duration	Participants had an average sleep duration of 492.2 ± 55.9 min. It was found that a negative correlation exists between disordered eating and sleep duration (ρ = −0.10)
(Khan et al., 2024) [26]	2024	40 European countries and the USA	Cross-sectional	Examine the relationship between excessive and problematic social media use and sleep problems among adolescents in 40 countries.	212,613 adolescents Average age of 13.52 (SD = 1.64) years, ranging from 11 to 15 years old 50.9% female and 49.1% male	4 item scale European Union Kids Online Survey, 9-item Social Media Disorder Scale, Likert question on the difficulty of falling asleep	Girls spent more time on social media than boys Problematic social media use predicted problems falling asleep both in girls (OR 2.20, 2.04–2.38) and boys (OR 1.88, 1.73–2.04). Heavy (frequent) social media use did not have this association
(Miedzobrodzka et al., 2024) [33]	2024	China and the Netherlands	Cross-sectional	Analyze associations between TikTok use and well-being, academic engagement, sleep quality, and bedtime procrastination in samples of high school and college students.	(471 total sample) 249 Chinese students in the adolescent sample Average age of 13.87 (SD = 0.85) years, range from 12 to 17 years 46.2% males and 48.2% females in the high school sample	Question about frequency of TikTok use and TikTok Self-Control Failure (TT-SCF) scale, 3 items from the Pittsburgh Sleep Quality Inventory (PSQI), and items about sleep procrastination due to social media	Uncontrolled and prolonged use of TikTok among high school students was associated with poorer well-being, (r TikTok use = 0.290), poorer academic performance (r TikTok use = 0.250), sleep quality (r TikTok use = 0.097; r TT-SCF = 0.127), and increased procrastination when it came to going to sleep (r TT-SCF = 0.532).
(Hà et al., 2023) [32]	2023	Vietnam	Cross-sectional	Examine the relationships between Facebook use and sleep quality, procrastination, life satisfaction, and self-compassion among Vietnamese students.	280 students Average age of 16.57 (SD = 0.0825) years, ranging from 15 to 17 64.6% female and 35.4% male	Facebook Addiction Scale (BFAS), Pittsburgh Sleep Quality Index (PSQI)	Facebook use was not associated with poorer sleep quality Self-compassion moderated the effect of excessive Facebook use and sleep quality
(Ali & Al-Shatari, 2023) [30]	2023	Bagdad, Irak	Cross-sectional	Assessing the impact of internet and social media use on the sleep of secondary school students	500 adolescents, most of whom were 16 years old (69.8%) 50.4% males and 49.6% females	Questionnaire with questions about time spent on social media and sleep quality	Most students went to bed between midnight and 2 a.m. (54.4%). Continuous use of social media throughout most of the day is associated with poorer sleep quality, fatigue, daytime sleepiness, and nightmares.
(Azhari et al., 2022) [34]	2022	East Anglia, UK	Cross-sectional	Analyze the association between the use of social media such as Facebook, Instagram, Twitter, and Snapchat and anxiety, loneliness, and sleep quality in girls aged 16 to 19.	41 female adolescents Average age of 17.83 (SD = 0.83) years, with an age range of 16 to 19 years	Questionnaire on social media use together with The Social Media Disorder Scale (SMD), Pittsburgh Sleep Quality Index (PSQI), and sleep diaries	27% of participants had problematic social media use and 68.3% had trouble limiting their use Facebook use was associated with poorer sleep quality. People with problematic social media use had poorer sleep frequency, especially on weekends
(Maksniemi et al., 2022) [38]	2022	Helsinki, Finland	Cross-sectional	Analyze the influence of social media use at bedtime and emotional exhaustion.	426 adolescents aged 13 to 19 65.7% women and 34.3% men	Questions about social media use and bedtime, Sociodigital Participation Inventory	Active use of social media was associated with delayed bedtimes only in adolescents aged 13 to 14. In late adolescence, going to bed later due to social media indicates greater emotional exhaustion.
(Chaveepojnkamjorn et al., 2021) [31]	2021	Thailand	Cross-sectional	Exploring sleep quality and identifying its association with social media use among adolescents in grades 10–12	777 students from eight schools Average age of 16.51 (SD = 0.96) years 70.39% female and 35.4% male	Sleep quality (Pittsburgh Sleep Quality Index PSQI) and assessment of social media content and technology use	Average social media use of 3.58 h per day 56.24% of adolescents had poor sleep quality Social media users had a 2.34 times higher risk of sleep problems
(Bergfeld & Van den Bulck, 2021) [35]	2021	New York, USA	Cross-sectional	Investigate the impact of social media usage habits on sleep quality, wakefulness, fatigue, and sleep duration in adolescents.	337 students from a high school in New York Average age of 15.7 years (SD = 1.17), range from 12 to 18 years old 54.3% female and 45.7% male	Network use questions, Bergen Facebook Addiction Scale, The Bed Time Shuteye Time measure, Pre-Sleep Arousal Scale, Pittsburgh Sleep Quality Index (PSQI) and The Fatigue Assessment Scale	Nighttime and/or problematic social media use led to poorer sleep quality Preference for Snapchat indicated a later bedtime, while Instagram did not have this effect
(Otsuka et al., 2021) [36]	2021	Japan	Cross-sectional	Examine the association between Internet usage time and sleep problems in Japanese adolescents	248,983 adolescents aged 12 to 18 years old	Questions about usage time and preferred social media platforms, questionnaire about insomnia symptoms, sleep duration, quality, and bedtime	Social media use was associated with going to bed later and poorer sleep quality Spending more than 5 h a day in front of a screen was associated with a higher likelihood of insomnia, poorer sleep quality, and a later bedtime
(van den Eijnden et al., 2021) [37]	2021	The Netherlands	Longitudinal	Understanding the longitudinal relationship between social media use and sleep in adolescents, as well as clarifying the role of parents in protecting against potential media influences	2021 adolescents Average age of 13.86 years, range of 11–17 years 54.6% males and 45.4% females	Questions about frequency of social media use, bedtime, Social Media Disorder Scale, Groningen Scale for sleep quality	More frequent and problematic social media use predicted a later bedtime in adolescents one year later (β = 0.07, *p* < 0.01). Strict parental rules about phone use before bedtime predicted an earlier bedtime
(Varghese et al., 2021) [27]	2021	Italy	Cross-sectional	Understanding the relationship between the frequency of technology and social media use and difficulties falling asleep among adolescents	3172 adolescents aged 11, 13, and 15 53% female and 47% male	Questions about the use of technology and social media, measuring the frequency of problems falling asleep	34.3% reported difficulty sleeping, which was more common among girls (39.2%) than boys (29.7%). Frequent use of technology and social media is associated with difficulty falling asleep in adolescents.
(Sümen & Evgin, 2021) [28]	2021	Turkey	Cross-sectional	Examine the relationship between social media addiction, sleep quality, and psychological problems in high school students.	1274 students with an average age of 15.36 years, ranging from 14 to 17 years old 70% female and 30% male	Social Media Addiction Scale for Adolescents (SMASA), Strengths and Difficulties Questionnaire (SDQ), and Sleep Variables Questionnaire (SVQ)	65.6% did not turn off their phones when sleeping, 42.8% placed them near their beds Problematic social media use was associated with emotional, behavioral, and attention problems, problems with peers, and poor sleep quality
(Evers et al., 2020) [25]	2020	Taiwan, China	Longitudinal	Measuring sleep quality in relation to the type of social media use at night and its relationship to academic engagement	2283 students, 52.5% male and 44.5% female Average age of 13.9 (SD = 0.72) years at T1 and 14.3 (SD = 0.66) years at T2	Disturbed Sleep Questionnaire (DSSM)	Only 20% of adolescents had sleep problems due to social media use (frequency of checking notifications and nighttime use). Poorer academic performance increased nighttime social media use.
(Makhfudli et al., 2020) [29]	2020	Surabaya, Indonesia	Cross-sectional	To determine the relationship between the intensity of social media use and sleep quality, social interaction, and self-esteem in urban adolescents.	141 adolescents, most of whom were 17 years old (58.2%) Aged between 15 and 18 63.1% male and 36.9% female	Questionnaire that included Social Networking Time Use (SONTUS) and Pittsburgh Sleep Quality Index (PSQI)	27% of adolescents who used social media heavily had very poor sleep quality. The more frequently they used social media such as YouTube, Instagram, Twitter, Facebook, or Path, the worse their sleep quality (*p* = 0.000; r = −0.459), self-esteem (*p* = 0.001; r = −0.286), and social interaction (*p* = 0.000; r = −0.348).

## Data Availability

The raw data supporting the conclusions of this article will be made available by the authors upon request.

## Supplementary Materials

The following supporting information can be downloaded at https://www.mdpi.com/article/10.3390/healthcare13222962/s1. Table S1: Summary of confounding control and cultural context across all included studies.

## Appendix A

### Appendix A.1. Complementary Tables

This material contains the detailed statistical output of the two partial random-effects meta-analyses described in Section 4.3. of the main manuscript.

**Table A1:** Results of the random-effects meta-analysis (Der Simonian–Laird method) for disordered eating (continuous outcomes).

Disordered Eating (Continuous Outcomes)						
Parameter	Estimate	95% CI Lower	95% CI Upper	95% PI Lower	95% PI Upper	*p*
Pooled effect (r)	0.354	−0.129	0.701	−0.129	0.701	0.067
τ (tau)	0.000	0.000	2.272	—	—	—
τ^2^ (tau-squared)	0.000	0.000	5.162	—	—	—
I^2^ (%)	0.000	0.000	99.898	—	—	—

Note. Correlations were Fisher z-transformed before analysis and back-transformed for interpretation.

**Table A2:** Results of the random-effect meta-analysis (Der Simonian-Laird method) for sleep quality (continuous outcomes).

Sleep Quality (Continuous Outcomes)						
Parameter	Estimate	95% CI Lower	95% CI Upper	95% PI Lower	95% PI Upper	*p*
Pooled effect (r)	0.355	0.186	0.505	−0.036	0.652	0.005
τ (tau)	0.131	0.072	0.433	—	—	—
τ^2^ (tau-squared)	0.017	0.005	0.187	—	—	—
I^2^ (%)	92.073	77.820	99.218	—	—	—

Note: The pooled correlation coefficient (r) was obtained under a Der Simonian–Laird random-effects model using Fisher’s z transformation.

### Appendix A.2. Complementary Figures

This material contains more detailed statistical output of the two partial random-effects meta-analyses described in Section 4.3. of the main manuscript.

This material forms an integral part of the main publication and follows the PRISMA 2020 reporting guidelines [15].
