# Supplementary material for: The Impact of Social Media on Adolescents’ Eating and Sleeping Habits: A Systematic Review and Meta-Analysis
Source: Healthcare (Basel). 2025 Nov 18;13(22):2962. doi: 10.3390/healthcare13222962 (PMC12652198; doi:10.3390/healthcare13222962)
Supplement: Supplementary file 1 [file healthcare-13-02962-s001.zip › healthcare-3947316-supplementary.pdf]

Supplementary Material – Table S1

This supplementary material contains a methodological synthesis of all studies included in the systematic review. Table S1 presents detailed information on:

- The outcome measurement instrument used in each study
- The country or cultural context
- Whether confounders were statistically controlled for in the analyses

This table complements *Section 3.5 (Quantitative synthesis)* and the *Limitations* subsection in the main manuscript by providing explicit transparency regarding heterogeneity in study design, analytical strategies, and statistical adjustment.

Supplementary Table S1. Summary of confounding control and cultural context across included studies.

Study (year)	Instrument / outcome evaluated	Country / cultural context	Confounders controlled (as reported in article)
**Jaruga-Sękowska et al., 2025**	Eating disorder risk (EAT-26), self-esteem (RSES), eating habits (MEH)	Poland	Regression; specific covariates NR
**Jeong & Shin, 2022**	Dietary satisfaction (SEM)	South Korea	SEM; covariates embedded (NR)
**Ryu et al., 2022**	Dietary risk factors; smartphone patterns	South Korea	Multivariate model; covariates NR
**Joo et al., 2024**	Unhealthy food consumption	South Korea	Adjusted logistic regression (sociodemographics, health behaviors)
**Zimmer-Gembeck et al., 2023**	Appearance preoccupation, disordered eating	Australia	Cross-lagged model incl. parental/peer influences
**Erdoğdu Yıldırım et al., 2025**	ED symptoms (EAT/EDES), body dissatisfaction	Türkiye	Regression mediation; covariates NR
**Livet et al., 2024**	Eating symptoms; mediation via self-esteem	Canada	Multilevel mediation; covariates NR
**Frieiro et al., 2022**	Eating disorder risk (EAT-26)	Spain	MANOVA/correlational; no confounder control

**Lonergan et al., 2020**	ED criteria (EDE-Q / DSM-5)	Australia	Multinomial logistic models; sex interaction
**López-Gil et al., 2024**	Disordered eating (SCOFF)	Spain	Adjusted (sex, BMI, waist, sleep time, SES)
**Evers et al., 2020**	Sleep disturbance due to SM use	NR	Cross-lagged model; covariates NR
**Khan et al., 2024**	Sleep difficulties; problematic SM use	40 countries	Multilevel mixed-effects regression & clustering
**Chaveepojnkamjorn et al., 2021**	Sleep quality (PSQI)	Thailand	Multivariable logistic regression (confounders not itemized)
**Hà et al., 2023**	Sleep quality; Facebook addiction	Vietnam	Serial mediation + moderation; covariates NR
**Miedzobrodzka et al., 2024**	Sleep quality; bedtime procrastination	Several countries	Multivariate model; covariates NR
**Azhari et al., 2022**	Sleep disturbances	United Kingdom	Regression; covariates NR
**Bergfeld & Van den Bulck, 2021**	Nighttime SM use → sleep indicators	USA	Regression including nighttime affordances
**Otsuka et al., 2021**	Insomnia; sleep onset	Japan	Weighted logistic model adjusting for grade, sex, breakfast, extracurricular activities, alcohol, smoking, school engagement, future education plans, mental health, survey year
**van den Eijnden et al., 2021**	Parental rules; SM use; sleep	Netherlands	Adjusted for age, gender, SES
**Maksniemi et al., 2022**	Bedtime; exhaustion; SM use	Finland	Random intercept longitudinal; covariates NR
**Varghese et al., 2021**	Sleep-onset difficulties	Italy	Logistic regression adjusted for demographic & behavioral factors
**Sümen & Evgin, 2021**	SM addiction; sleep quality	Türkiye	Path analysis (no classical confounder adjustment)
**Makhfudli et al., 2020**	Sleep quality	Indonesia	Bivariate; no confounder control

**Ali & Al-Shatari, 2023**	Sleep quality	Iraq	Regression; covariates NR

Note. NR = Not reported. "Confounders controlled" refers to explicit mention of covariate adjustment in the statistical model. Studies not reporting confounder adjustment were classified accordingly.
